# Editorial: The outbreak and sequelae of the increase in opioid use in the United States, Canada, and beyond

**DOI:** 10.3389/fsoc.2022.1023531

**Published:** 2022-09-05

**Authors:** Samuel R. Friedman, David C. Perlman, Ralph J. DiClemente

**Affiliations:** ^1^NYU Grossman School of Medicine, New York, NY, United States; ^2^Infectious Diseases, Mount Sinai Beth Israel, Icahn School of Medicine at Mount Sinai, New York, NY, United States; ^3^Department of Social and Behavioral Sciences, School of Global Public Health, New York University, New York, NY, United States

**Keywords:** opioid, overdose, despair, intervention, prevention

In 2018, there were 67,367 drug overdose deaths in the United States, Unfortunately, by 2021, the latest year for which data are available, the number of deaths had increased to over 107,000 deaths. This increase in overdose mortality was probably driven by a combination of upstream processes, most of which derive from ways in which capitalism and its system of nation-states are creating economic and cultural crises. These crises include the COVID-19 pandemic, economic crises, and a deepening culture of despair (Friedman et al., 2021). Relatedly, the illicit drug markets for stimulants and opioids have changed and have come to include widespread highly-potent synthetic opioids (Baldwin et al., 2021). Articles in this special issue provide insights into existing and potential strategies to prevent risky opioid use and reduce opioid mortality. We briefly discuss each of the articles in this issue and highlight key ideas, constructs, and recommendations for research and intervention.

Friedman et al. present evidence that the opioid/overdose epidemic is not only a question of individual behaviors—although those are important—nor only of corporate greed in the over-zealous marketing of dangerous opioids as harmless pain medicines, but that the overdose epidemic is part of a deeper dialectic of one-sided class war, the impacts of economic trends on profits, wages, employment, wealth and housing inequality, and of the associated social, community, ideological and psychological changes these cause.

The recent changes in the racial/ethnic distributions of overdose mortality suggest that the processes discussed in the Friedman et al. paper have taken place in a deeply racialized society where economic, political, and ideological changes are shaped by, and in turn shape, patterns of oppression and of struggle (Friedman et al., 2022b).

Bergo et al. extend prior work by Van Handel et al. (2016) using area-level measures of several syndemically related processes to predict the *need* for overdose, HIV, and hepatitis C prevention. Lyss et al. present evidence that the CDC county vulnerability index has not been an effective predictor of HIV outbreaks. Bergo et al. add new measures to create a revised index and examine ecologic associations at the ZIPCODE rather than county level, permitting greater geographic precision. One research question raised by this paper is whether indicators of a locality's “need” for overdose interventions are actually associated with whether interventions are implemented and/or their scale. Research on the placement and magnitude of syringe service and drug treatment programs across metropolitan areas suggests that associations between “program need” and “program implementation” have been weak at best (Friedman et al., 2007; Tempalski et al., 2007, 2008). Similarly, further research is needed on whether localities that need overdose programs the most are those where interventions are likely to be most effective. Recent experience in the United States, where some States or other localities with particularly severe COVID-19 epidemics have rejected mask mandates (and have had a low response to voluntary masking) and/or have responded poorly to vaccination campaigns exemplify that need may not predict either the existence or effectiveness of programmatic responses (Kahane, 2021; Kelman, 2021; Sehgal et al., 2022).

Gaps between need and effective response may also interact with programs of stigmatization of people who use drugs, particularly since drug policy has long been racialized in the United States and this is likely to interact with trends for overdose mortality to become more associated with racially-oppressed minorities (Friedman et al., 2022a; Kiang et al., 2022; Townsend et al., 2022).

Treatment for opioid use disorder, particularly *medications for opioid use disorder (MOUD)* in the United States is sometimes pointed to as the key route to ending opioid-related overdoses and related mortality. Such suggestions, however, seem incomplete given the currently-limited population-level effectiveness of MOUD. As discussed in Williams et al. (2019), OUD treatment is inadequate in the US: Of the 2.1 million people who are estimated to need care, approximately 20% are receiving treatment, and only 35% of these are receiving FDA-approved medications (methadone, buprenorphine, extended-release naloxone). Furthermore, retention in treatment programs for 6 months or more is low, and long-term “remission” is even lower. Thus, getting enough people at risk for overdose into treatment will require a large increase in available treatment and an increase in the proportion who receive evidence-based treatment (MOUD). Once in treatment, however, the prospects for retention are low, and for a cure even lower. Thus, to have a substantial impact on the opioid/overdose crisis, treatment would need large increases in the number, geographic distribution, insurance coverage, quality, patient satisfaction, retention, and overall improvements in efficacy and population-level effectiveness.

In sum, then, the articles on treatment in this special issue by Blazes and Morrow, Mistler et al., and Frank and Walters offer useful, though insufficient, contributions to improving the opioid/overdose crisis. Population-level improvements will likely require implementing “upstream” interventions plus effective community-level interventions.

Blazes and Morrow address the co-formulation of buprenorphine and naloxone. The rationale for co-formulating these agents was to prevent the diversion of buprenorphine prescribed as MOUD to illicit injection use. Including naloxone (an opioid antagonist) with buprenorphine blunts the opioid effects, possibly (perhaps probably) reducing overdose and, by reducing euphoria when injected, possibly reducing incentives for diversion. The authors point out that this formulation has not consistently deterred its diverted use or misuse; this is confirmed by the observation that injection of buprenorphine-naloxone formulations is prevalent and, in some jurisdictions, is the most prevalent form of illicit drug injection (Johnson and Richert, 2019). Further data on the impact of this co-formulation on population-level overdose rates are needed.

Mistler et al. highlight that cognitive dysfunction, of various etiologies, can prevent achieving effective intervention outcomes to address the harms of opioid use. They suggest that for PWUD who enter methadone treatment, it is important to develop more effective ways of recognizing and addressing mental health disorders Based on two focus groups with providers and patients from one MMTP, they suggest ways to achieve this.

Frank and Walters conducted qualitative research with MOUD patients and treatment providers and showed that many patients enter MOUD not because they want to, but rather because they experience constrained choices attributable to drugs' illegality; peer and family pressure; fear that authorities seize custody of their children; and/or because of internalized stigma. Analyses of patients and their interaction with providers, however, often assume that patients are in treatment voluntarily, and treatment decisions are often made on that basis. Frank and Walters suggest that recognizing the often-coercive context of treatment-seeking may provide insights for providers and people in treatment to develop more productive interaction strategies. Further research is necessary to assess whether improved interaction results in reducing overdose mortality in the absence of changing the broad upstream, oppressive context.

Other papers in this issue lay the basis for community-level interventions. Some of these, such as Bagchi et al., Riazi et al., and Des Jarlais et al., approach this through community education and/or counseling. Ellis et al. suggest both educational interventions and changing the ways authorities respond to PWUD.

Bagchi et al. view opioid overdose, hepatitis C, and HIV as a syndemic that requires integrated interventions that incorporate consideration of each condition, and also suggest the need for interventions addressing broader underlying forces that increase risk; such as stigma, structural vulnerability, and siloed systems of care. They describe a 90-min Structural Competency Curriculum to train primary care providers. The limit of 90 min is realistic, since US health care focuses on high-profit individual care, even though it is widely recognized that addressing ‘upstream' social and structural determinants is crucial. Provider-level interventions are also self-limited. At best, they lead to better awareness and practice in patient-provider interactions, but this cannot change in oppressive structures that underlie the opioid epidemic.

Riazi et al. describe an innovative program to provide overdose education and naloxone distribution training to at-risk populations and bystanders so that overdoses can be reversed. The program was implemented at public events, community-based organizations, substance use programs, educational facilities, homeless prevention programs, faith-based organizations, and alternatives to incarceration programs. It also used a train-the-trainer model to teach medical students and nurses to train others in these techniques. This article also provides useful information about how they adapted this program during the COVID-19 initial emergency period.

Des Jarlais et al. present a model of how some people who inject drugs come to initiate other PWUD into injecting (which is associated with a higher risk of infections and overdose). The stages in this process are promulgating positive visions of injection drug use; being asked to initiate by someone, and then initiating. It has long since been proposed that harm reduction efforts might work with potential initiators to keep them from initiating others or, at least, convince them to model safer injection techniques (Hunt et al., 1998). Des Jarlais and his collaborators have developed a “Break the Cycle” intervention to locate likely initiators and train them not to initiate others. This intervention seems to reduce the extent to which such initiators initiate others into injection (Des Jarlais et al., 2019; Uusküla et al., 2022).

This is a promising intervention, but several important questions remain to be answered: 1. Do those PWUD who ask to be initiated find other people to initiate them? 2. Of those who do not, how many initiate without the assistance of an experienced injector? 3. Does the experience of being refused by a potential injector, or of being unable to find one, reduce the subsequent probability of overdosing, dying from an overdose, or becoming infected among PWUD who asked? 4. Does implementing the Breaking the Cycle intervention in a locality or in a social network of PWUD reduce the rate of initiating injection and/or overdose in that locality or network?

Ellis et al. studied the healthcare experiences of PWUD in rural Southern Illinois qualitatively. Participants reported several ways in which their treatment dissuaded them from using medical services. These included forced catheterization, divulging drug test results to law enforcement, sharing details of counseling sessions with community members, and fear of calling emergency services if someone had an overdose. They suggest reforming and clarifying law enforcement's role in Emergency Departments, instituting diversion policies during arrests, stigma training, and harm reduction education for emergency medicine providers, and referral systems between Emergency Departments and local harm reduction agencies. These suggestions have some basis in practical experience and in theory, but research is needed to see if they can restore PWUD's trust in medical services and, in particular, if such efforts can reduce fatal overdoses.

Ventuneac et al. and Guarino et al., focus on the epidemiology of risk.

Ventuneac et al. show that people living with HIV disproportionately use opioids. To some extent, this may be attributable to HIV acquisition through high-risk injection or sexual practices. In addition, some people living with HIV have had periods of severe pain due to HIV-associated complications or morbidities, which may lead to drug initiation, dependency, and overdose risk.

Guarino et al. studied a group of community-recruited young adult (aged 18–29) opioid users to assess the association of childhood traumatic events with the age of initiation of seven different drug behaviors. They observed that the more types of childhood traumatic events participants experienced, the earlier the age at which they underwent each kind of drug use initiation. This suggests that childhood trauma may contribute to vulnerability to high-risk drug use. A cohort study could provide additional information about these relationships.

What is not clear, in the context of 40 years of increasing overdose mortality in the United States (Jalal et al., 2018), which during some periods has been closely tied to increasing opioid use, is whether the increase in opioid use and/or overdose mortality at the population level is, in part, caused by increases in childhood trauma. It is certainly plausible, for example, that the one-sided class war described by Friedman et al. could engender family and individual stressors among adults that would, in turn, lead to increases in childhood trauma. Greater understanding of this pathway, and its prevalence, may point the way to developing innovative prevention methods that intervene against overdose mortality by an upstream approach to reducing childhood trauma.

Many of the research and innovative proposals discussed in these papers concern upstream interventions or expanding and improving existing harm reduction and treatment efforts. Although no papers focused on these, we would also suggest ensuring a safer drug supply may reduce the overdose risks from synthetic opioid adulterants. Additionally, repealing the criminalization of drug use may lead to greater drug treatment seeking, reduced stigma, and, as a consequence, less opioid-associated mortality. Given the scope of the opioid crisis, we would urge expanded effort to develop, implement, and evaluate innovative strategies, community partnerships, and public health policies.

## Author contributions

All authors listed have made a substantial, direct, and intellectual contribution to the work and approved it for publication.

## Funding

This work was supported by the United States National Institute on Drug Abuse grant P30 DA011041.

## Conflict of interest

The authors declare that the research was conducted in the absence of any commercial or financial relationships that could be construed as a potential conflict of interest.

## Publisher's note

All claims expressed in this article are solely those of the authors and do not necessarily represent those of their affiliated organizations, or those of the publisher, the editors and the reviewers. Any product that may be evaluated in this article, or claim that may be made by its manufacturer, is not guaranteed or endorsed by the publisher.

## Author disclaimer

The content is solely the responsibility of the authors and does not necessarily represent the official views of the National Institutes of Health.

## References

[B1] Baldwin G. T. Seth P. Noonan R. K. (2021). Continued increases in overdose deaths related to synthetic opioids: implications for clinical practice. JAMA. 325, 1151–1152. 10.1001/jama.2021.1169 33571368 PMC9795485

[B2] Des Jarlais D. Uuskula A. Talu A. Barnes D. M. Raag M. Arasteh K.. (2019). Implementing an updated “break the cycle” intervention to reduce initiating persons into injecting drug use in an eastern european and a us “opioid epidemic” setting. AIDS Behav. 23, 2304–2314. 10.1007/s10461-019-02467-y 30879209 PMC6746607

[B3] Friedman S. R. Mateu-Gelabert P. Nikolopoulos G. K. Cerdá M. Rossi D. Jordan A. E.. (2021). Big Events theory and measures may help explain emerging long-term effects of current crises. Glo? Public Health 16, 1167–1186. 10.1080/17441692.2021.1903528 33843462 PMC8338763

[B4] Friedman S. R. Tempalski B. Brady J. E. Friedman J. J. Cooper H. L. Flom P. L.. (2007). Predictors of the degree of drug treatment coverage for injection drug users in 94 metropolitan areas in the United States of America. Int. J. Drug Policy 18, 475–485. 10.1016/j.drugpo.2006.10.004 18061873

[B5] Friedman S. R. Williams L. D. Guarino H. Mateu-Gelabert P. Krawczyk N. Hamilton L.. (2022a). The stigma system: How sociopolitical domination, scapegoating, and stigma shape public health. J. Community Psychol. 50, 385–408. 10.1002/jcop.22581 34115390 PMC8664901

[B6] Friedman S. R. Williams L. D. Jordan A. E. Walters S. Perlman D. C. Mateu-Gelabert P.. (2022b). Toward a theory of the underpinnings and vulnerabilities of structural racism: looking upstream from disease inequities among people who use drugs. Int. J. Environ. Res. Public Health 19:7453. 10.3390/ijerph19127453 35742699 PMC9224240

[B7] Hunt N. Stillwell G. Taylor C. Griffiths P. (1998). Evaluation of a brief intervention to prevent initiation into injecting. Drugs 5, 185–194. 10.3109/09687639809006684

[B8] Jalal H. Buchanich J. M. Roberts M. S. Balmert L. C. Zhang K. Burke D. S.. (2018). Changing dynamics of the drug overdose epidemic in the United States from 1979 through 2016. Science 361:aau1184. 10.1126/science.aau1184 30237320 PMC8025225

[B9] Johnson B. Richert T. (2019). Non-prescribed use of methadone and buprenorphine prior to opioid substitution treatment: lifetime prevalence, motives, and drug sources among people with opioid dependence in five Swedish cities. Harm. Reduct. J. 16:31. 10.1186/s12954-019-0301-y 31046774 PMC6498489

[B10] Kahane L. H. (2021). Politicizing the mask: political, economic and demographic factors affecting mask wearing behavior in the USA. East Econ. J. 47, 163–183. 10.1057/s41302-020-00186-0 33424048 PMC7783295

[B11] Kelman B. (2021).*Tennessee Fires Top Vaccine Official as, COVID-19 Shows Signs of New Spread*. The Tennessean.

[B12] Kiang M. V. Acosta R. J. Chen Y. H. Matthay E. C. Tsai A. C. Basu S.. (2022). Sociodemographic and geographic disparities in excess fatal drug overdoses during the COVID-19 pandemic in California: A population-based study. Lancet Reg. Health Am. 11:100237. 10.1016/j.lana.2022.100237 35342895 PMC8934030

[B13] Sehgal N. J. Yue D. Pope E. Wang R. H. Roby D. H. (2022). The association between COVID-19 mortality and the county-level partisan divide in the United States. Health Affairs 41, 853–863. 10.1377/hlthaff.2022.00085

[B14] Tempalski B. Cooper H. L. Friedman S. R. Des Jarlais D. C. Brady J. Gostnell K.. (2008). Correlates of syringe coverage for heroin injection in 35 large metropolitan areas in the US in which heroin is the dominant injected drug. Int. J. Drug Policy 19, S47–S58. 10.1016/j.drugpo.2007.11.011 18295468 PMC2706511

[B15] Tempalski B. Flom P. L. Friedman S. R. Des Jarlais D. C. Friedman J. J. McKnight C.. (2007). Social and political factors predicting the presence of syringe exchange programs in 96 US metropolitan areas. Am. J. Public Health 97, 437–447. 10.2105/AJPH.2005.065961 17267732 PMC1805016

[B16] Townsend T. Kline D. Rivera-Aguirre A. Bunting A. M. Mauro P. M. Marshall B. D. L.. (2022). Racial/ethnic and geographic trends in combined stimulant/opioid overdoses, 2007-2019. Am. J. Epidemiol. 191, 599–612. 10.1093/aje/kwab290 35142341 PMC9077116

[B17] Uusküla A. Raag M. Barnes D. Tross S. Talu A. Des Jarlais D.. (2022). Adapted “break the cycle for avant garde” intervention to reduce injection assisting and promoting behaviours in people who inject drugs in tallinn, estonia: a pre- post trial. medRxiv 29:22273126. 10.1101/2022.03.29.22273126 PMC10231841 37256867

[B18] Van Handel M. M. Rose C. E. Hallisey E. J. Kolling J. L. Zibbell J. E. Lewis B.. (2016). County-level vulnerability assessment for rapid dissemination of HIV or HCV infections among persons who inject drugs, United States. J. Acquir. Immune. Defic. Syndr. 73, 323–331. 10.1097/QAI.0000000000001098 27763996 PMC5479631

[B19] Williams A. R. Nunes E. V. Bisaga A. Levin F. R. Olfson M. (2019). Development of a cascade of care for responding to the opioid epidemic. Am. J. Drug Alcohol. Abuse 45, 1–10. 10.1080/00952990.2018.1546862 30675818 PMC6404749

